# A scoping review of augmented reality in nursing

**DOI:** 10.1186/s12912-019-0342-2

**Published:** 2019-05-16

**Authors:** Hanna Wüller, Jonathan Behrens, Marcus Garthaus, Sara Marquard, Hartmut Remmers

**Affiliations:** School of Human Sciences, Osnabrück University, Osnabrück, Lower Saxony Germany

**Keywords:** Nursing, Augmented reality, Evaluation methods, Values, Scoping review

## Abstract

**Background:**

Augmented reality (AR) has the potential to be utilized in various fields. Nursing fulfils the requirements of smart glass use cases, and technology may be one method of supporting nurses that face challenges such as demographic change. The development of AR to assist in nursing is now feasible. Attempts to develop applications have been made, but there has not been an overview regarding the existing research.

**Objective:**

The aim of this scoping review is to provide an overview of the current research regarding AR in nursing to identify possible research gaps. This led to the following research question: “To date, what research has been performed regarding the use of AR in nursing?”. A focus has been placed on the topics involving cases, evaluations, and devices used.

**Methods:**

A scoping review was carried out with the methodological steps outlined by Arksey and O’Malley (2005) and further enhanced by Levac et al. (2010). A broad range of keywords were used systematically in eight databases including PubMed, Web of Science and ACM to search for topics in nursing.

**Results:**

The search led to 23 publications that were included in the final analysis. The majority of the identified publications describe pilot studies. The methods used for identifying use cases and evaluating applications differ among the included studies. Furthermore, the devices used vary from study to study and may include smart glasses, tablets, and smart watches, among others. Previous studies predominantly evaluated the use of smart glasses. In addition, evaluations did not take framing conditions into account. Reviewed publications that evaluated the use of AR in nursing also identified technical challenges associated with AR.

**Conclusions:**

These results show that the use of AR in nursing may have positive implications. While current studies focus on evaluating prototypes, future studies should focus on performing long-term evaluations to take framing conditions and the long-term consequences of AR into consideration. Our findings are important and informative for nurses and technicians who are involved in the development of new technologies. They can use our findings to reflect on their own design of case identification, requirements for elicitation and evaluation.

## Background

In recent years, there has been an increase in the development of innovative digital technology. One recent technology is Augmented Reality (AR). AR is the enhancement of reality with virtual content [1].

AR offers a wide range of possible uses [2]. Overviews regarding these cases have been published in various fields including construction [3], educational settings [4, 5], manufacturing and design [6, 7] and marketing [8]. In the field of healthcare, studies show potential for the use of AR in surgical applications and medical education [9–16].

Cases supporting the use of smart glasses exist if an application is needed to be timely, mobile, and hands-free and continuous attention on the task is necessary [2]. Nursing is an interesting field in which to apply AR as these characteristics are applicable to many tasks in the nursing field. Furthermore, demographical change and rising multimorbidity are challenges addressed by nurses [17]. Technical solutions and social innovation may improve healthcare; however, it is important to take the special circumstances of care workers into consideration [18, 19].

Cases supporting the use of AR in nursing were examined in the research project Augmented Reality in flexible service processes (ARinFLEX) that discusses the topic of AR in the fields of maintenance and nursing. Another project (Pflegebrille) follows the goal of making smart glasses usable for ambulatory healthcare.

Furthermore, the use of technology in nursing is increasing [20]. The aim of this scoping review is to give an overview of existing research on the use of AR in nursing to identify research gaps and provide information for future studies with the following research question:

- *To date, what research has been performed regarding the use of AR in nursing?*

## Methods

We performed a literature review using a scope study methodology because the use of AR in nursing has not been well studied. Scope studies are useful “especially where an area is complex or has not been reviewed comprehensively before” [21]. Scoping studies may be particularly relevant to disciplines with emerging evidence such as the use of AR in nursing [22]. In contrast to systematic reviews, scope studies work with a broad research question and forego a quality assessment [22–24]. Even though “no universal agreement exists on terminology, definition or methodological steps” [25], we followed the framework by Arksey & O‘Malley, who developed key phases for scoping studies [24]. In 2010, Levac et al. refined the framework and released a variety of methodical recommendations [22]. The following steps have been particularly useful for us: (1) using a broad research question with a clear definition of the purpose for our study, (2) selecting and abstracting data by an iterative, team-oriented approach, and (3) identifying themes and charting the data (ibid).

### Data sources and searches

To answer our research question, we carried out a scoping review using a systematic search of the databases PubMed, CINAHL, PsycINFO, Web of Science Core Collection, Cochrane, ACM and AISEL. We chose databases from the fields of healthcare and technology as well as interdisciplinary databases to provide relevance for our research question.

We used the keywords ‘Nursing’, ‘Care’ OR ‘Caring’ in combination with the phrases ‘Augmented Reality’, ‘AR device’, ‘AR glass’, ‘Smart device’, ‘Smart glass’, ‘Smart watch’ OR ‘Google glass’. Truncations were used where appropriate. We used the terms ‘Smart device’, ‘Smart glass’, ‘Smart watch’ and ‘Google glass’ to include studies that describe AR applications but do not name them as one. The phrase “Google Glass” was chosen because it is one of the most prevalent, commercially available smart glasses. Furthermore, the term ‘Smart Devices’ is not limited to ‘Smart Glasses’ or ‘Smart Watches’. The final search strategy for PubMed was: (nurs* OR care OR caring) AND (“augmented reality” OR “smart glass” OR “smart watch*” OR “smart device” OR “google glass” OR “augmented reality glasses” OR “AR glass” OR “AR device”). The search was conducted on April 9th, 2018 and no Limits were used.

We also checked the bibliographies of each study and used existing networks and organizations to identify additional relevant studies [24]. These networks consisted of experts in the fields of Business Computer Science and Nursing. Due to resource limitations, we did not perform a hand-search of key journals. Hits in English or German were considered. Any date of publication was acceptable. The reference manager tool EndNote was used to compile relevant literature and to identify duplicates.

### Study selection

**Fig. 1:**
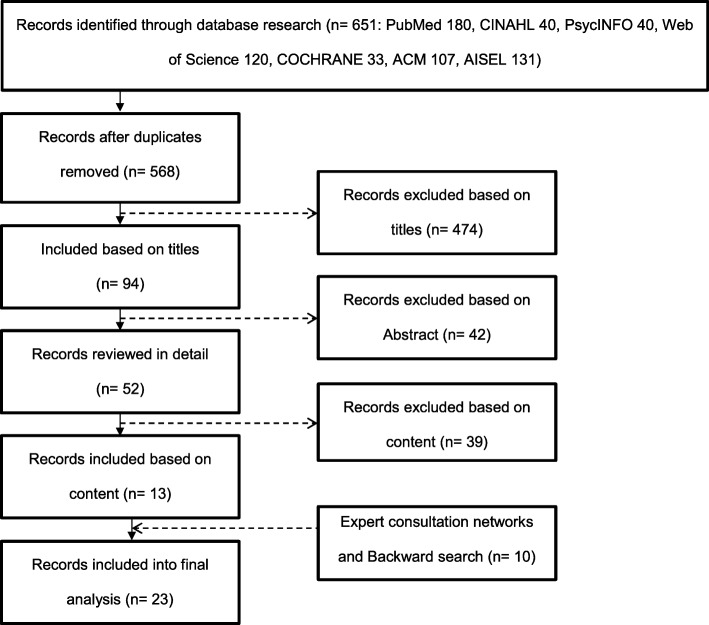
Flow diagram depicting the study selection process

This study used the PRISMA-ScR Checklist which consist a flow diagram. The flow diagram (Fig. 1) allows a transparent reporting of the literature findings based on conceptual and practical advances in the science of systematic reviews [26]. Author one (HW, nursing scientist; M.Sc. Public Health, B.Sc. Business Computer Science) and author two (JB, student of nursing science) reviewed the titles and abstracts using the inclusion criteria (Table 1). Only articles written in English and German were included because of the authors linguistic background. Articles chosen by both reviewers were automatically included. Articles chosen by one reviewer were audited by a third independent reviewer (MG, gerontologist in the field of nursing in technology; M.A. Health Care Management, B.A. Gerontology). The full text of the remaining publications was then reviewed.

**Table 1:** Inclusion and exclusion criteria

	Inclusion	Exclusion
Language	German, English	Non-English and Non-German
Time Period	Studies published until April 2018	Any Study after April 2018
Study focus	AR or smart glasses used to support nurses in their work or in education	Any Study not mentioning nurses/ nursing; Any Study mentioning usage of AR only through patients
Study Design	Any	Nil
Setting	Any	Nil

## Data extraction

The articles selected for a full-text review were charted by JB, HW, and MG [22, 24]. A mind map was drawn by HW to collect emerging topics. The mind map was audited by HW, JB, and MG to identify the most prominent and relevant categories and to support the challenging process of charting the data [22].

We then iteratively developed the charting form and included relevant, emerging topics in the form with the details of each article. Charting was performed in parallel by two reviewers for a sample of articles. Once the charting process was completed, we synthesized the results to develop summary findings pertinent to the variables in the charting form. We then considered these summary findings in the context of AR in nursing in order to develop recommendations for future research, which was consistent with the stated purpose of our review [22].

## Results

No manuscripts published earlier than 2007 were detected. Five of the studies used qualitative methods, six used quantitative methods, and nine used mixed methods. Eleven studies were published by authors from Europe, nine studies were published by authors from America, two studies were published by authors from Asia and one study was published by authors from Australia. For three studies, the method is not clearly described. The studies that were identified are displayed in detail in Table 2. The results of the relevant topics are described in the following subsections.

**Table 2:** Details of included studies

No.	Author Year Country	Study type and Study objective	Methods a) Use case identification b) Requirements elicitation c) Evaluation	Use case description(s)	Device used Device, Technical challenges	Major findings
1	Aldaz et al. 2015 USA	- Pilot Study, mixed methods - Presenting development and assessment of the SnapCap System for chronic wound photography	a) Shadowing, interviews (*n* = 16) c) 10–15-min follow-up session and following post-task questionnaire (*n* = 16)	- Wound care management with smart glasses	- GG - Network connectivity - Speech recognition	- Hands-free device considered beneficial; - Barcodes and speech recognition are positive aspects; - To be considered further: Privacy, camera resolution, speech recognition.
2	Byrne 2017 USA			- Patient’s veins to be more readily visible - Tools for emergency preparedness and for a wide range of difficult-to-simulate training situations	- Immersion sickness - Over-reliance on technology - Lack of attention to surroundings	- Exploration of AR/ VR for anxiety and pain control will increasingly - have relevance for perianesthesia nurses.
3	Byrne et al. 2017 USA	- Pilot study, mixed methods - Evaluate students’ perceptions related to their experience using GG	c) Survey that contained 9 questions that used a 4-point Likert scale and two open-ended narrative questions (*n* = 11)	- Information retrieval and communication in teaching	- GG	- GG overall helpful; - Benefits: Time savings, easy information retrieval; - To be considered further: Nurse may focus on the device instead of the patient.
4	Ehrler et al. 2015 Switzerland	- Pilot study, qualitative methods - Presenting a solution enabling the display of care protocols through GG	b) User centred design, interviews and observations (*n* = 3)	- Intravenous injection of a drug to a patient - Step by step guidance	- GG; - Autofocus; - Screen resolution; - Voice recognition	- Valuable experience about the use of GG for the display of guidelines in healthcare settings; - GG usage overall positive.
5	Ehrler et al. 2016 Switzerland	- Qualitative study - Presenting transformation of clinical guidelines into a representation that can be used on GG	b) Focus group and observations (*n* = 3)	- Development of guidelines to display the pediatric cardiac arrest algorithm for support to provide guidelines at point of care	- GG, - Screen size	- Guidelines are developed; - Next step: Guidelines have to be evaluated.
6	Frost et al. 2017 (Australia)			- Nursing education;	- HoloLens	- Can be used to guide clinical assessment as a means to integrate knowledge to formulate plans of care and develop clinical reasoning skills.
7	Fumagalli et al. 2016 Italy	- Pilot study; quantitative methods - Comparison of efficacy and safety of Near-infrared electromagnetic radiation based devices with the standard technique in elderly patients	c) Mini Mental State Examination, Visual Analogue Scale, Hospital Anxiety and Depression Scale (*n* = 103)	- Intensive care unit; - Support of blood sampling with tablet application to visualize veins	- Novel devices based on the emission of near-infrared electromagnetic radiation; - No technical challenges described	- No difference in number of attempts and time; - Lower anxiety and depression of the patient.
8	Garrett et al. 2015 Canada	- Pilot study, mixed methods - Exploration if new mobile AR technologies have the potential to enhance the learning of clinical skills in the lab	c) Online evaluation questionnaire (*n* = 72), focus group interviews (*n* = 6)	- Clinical lab equipment explanations through usage of bar codes/QR codes - AR resources were integrated into a clinical simulation scenario	- Smartphones and Tablets (iPad) - Response times; - Incompatible smartphones; - Scanning; - Internet connection; - Stability of application	- Use of AR demonstrated some potential; - Further integration and evaluative work warranted.
9	González et al. 2014 Mexico	- Pilot study, quantitative methods - Propose smart multi-level tool for remote patient monitoring	c) Comparative trials (*n* = 10; 50 diagnoses)	- Remote monitoring of body temperature and heart rate by wireless sensor network and mobile AR	- Arduino microcontroller; - PCs; - Smartphones - Sensors	- Decreased time needed to monitor patients; - Automatic diagnosis in real time - Remote alarm generation; - Generation of virtual files.
10	Grünerbl et al. 2015 UK	- Pilot study, quantitative methods - Monitoring and Enhancing Nurse Emergency Training with Wearable Devices	c) Evaluation of recorded localization data by an expert; not further specified (*n* = 7)	- Augment training scenario to give better feedback to learners	- GG, Smart Watch;	- Significant amount of information about relevant activity and cooperation patterns is contained in the data; - Further research necessary.
11	Klinker et al. 2017 Germany	- Pilot study, mixed methods - Presenting a preliminary design	b) Design Science Research Method (three iterations); System Usability Scale + open questions + verbal comments (*n* = 39; *n* = 9; *n* = 14)	- Serious game to improve hand hygiene	- Microsoft HoloLens	- Presentation of a novel approach by employing serios game with paralles to health care workers daily routine
12	Kopetz et al. 2018 Germany	- Quantitative study - Presentation of method and results	a) Online survey (*n* = 107)	- Practical education of nurses; Scenario: transfer from bed to wheelchair		- AR may have advantages for nursing education (individuality, vizualization); - (Potential) Users must be convinced gradually.
13	Mentler et al. 2016 Germany	- Qualitative study - Discussing optical head-mounted displays with respect to humancomputer interaction	a) Literature review and interviews (> 25) c) Observation and interviews 1) *n* = 14; 2) *n* = 14; 3) *n* = 12; 4) *n* = 2)	- Supporting triage process in mass casualty incidents - Identifying dangerous goods - Coordinating duration of infusions - Picture based documentation of surgery Device: GG	- GG - Image quality	- Great interest in optical smart glasses; - Efficient and safe usage seems possible; - Current workflows can be improved; - To be improved: Technical reliability and features (e.g. camera quality); Attention is needed to perform hands-free interaction with an application.
14	Nilsson & Johansson 2007 Sweden	- Mixed methods study - Discussing usability and user acceptance aspects of an AR system from a Cognitive Systems Engineering perspective	c) Observations and quantitative questionnaire (*n* = 12)	- AR instructions on how to assemble a common medical device	- Head Mounted Display; - Marker problems	- Users are positive towards AR systems for instructions; - AR may become an accepted part of everyday work; - System is fun to use; - Possibility to get objective information in an easy way
15	Nilsson & Johansson 2008 Sweden	- Pilot study, mixed methods - Test AR in real world scenarios	b) Instructions received were developed with the model of an operating room nurse c) Observation (*n* = 8) and open ended questionnaires (*n* = 12)	- Instructions on how to interact with a diathermy apparatus - Instructions how to assemble a trocar Device: Helmet Mounted Display	- Helmet Mounted Display (Sony Glasstron); - Bulky helmet; - Marker problems; - Parallax vision	- Interactivity seems to be important for an AR system; - Users would prefer the possibility to ask the system random questions; - Objectivity of instructions made by the system was mentioned positively.
16	Pugoy et al. 2016 Philipines and Thailand	- Pilot study, quantitative methods - Provide a proof of concept for budget constrained and technologically challenged implementers	c) SUS + 3 additional questions (*n* = 17)	- Improve the English communication skills of nursing professionals	- Mobile device	- AR can be used by budget constrained and technologically challenged implementers from developing countries
17	Rahn & Kjaergaard 2014 Denmark	- Mixed method study - Investigation of potentials of AR as an educational technology.	c) Filmed processes analyzed through meaning condensation (n =?); evaluative questionnaires (*n* = 14)	- AR in the teaching of highly complex anatomical and physiological subjects in the training of nurses at undergraduate level	- iPad, - APP has to be dependable	- The use of AR does appear to have the potential to facilitate student learning and increase their level of understanding of the subject matter at hand; - Students can see potential in the use of AR in their future education.
18	Rochlen et al. 2017 USA	- Pilot study, mixed methods - Evaluating usability and feasibility	c) Survey describing their perceptions (*n* = 40)	- A 1st person point of view AR trainer on needle insertion	- Epson Moverio BT-200® Smart Glasses	- First person point of view AR technology is a potentially promising training tool for central line placement.
19	Samosky et al. 2012 (USA)	- Prototype description - Present novel features of the Body ExplorerAR platform		- Education for healthcare, enhance mannequin with additional information	- Projector, Wiimote, IR Light Pen	- Provides a testbed for AR enhancements.
20	Schneidereith 2015 USA	- Qualitative study - Describe errors in medication administration identified through usage of GG	c) Review of GG videos; Method is not described (*n* = 10)	- Observation of students when performing medication tasks through their perspective to identify mistakes	- GG, - Network connectivity	- Identification of mistakes made by students is easy and can be used to improve teaching plans.
21	Vaughn et al. 2016 USA	- Pilot study, Mixed methods study - Describing the pilot study	c) 2 experts evaluated students skill + survey based on Simulation Design Scale and Self-Confidence in Learning scale (*n* = 15)	- Project video into students’ vision to increase the perception of realism	- AR Headset, - Connectivity issues, - Lack of experience with system, - Battery life	- Using the device supported simulation; - The simulation gave the students confidence; - Barriers were related to lack of experience with the device; - Due to the concentration on the system other hints may be missed; - 80% of the students would recommend using the technology.
22	Yoshida et al. 2015 Japan	- Pilot study, quantitative methods - (Prospective) Evaluation of the usefulness of seethrough–type head-mounted display as a novel intraoperative instructional tool for scrub nurses.	c) Self-made questionnaire (*n* = 15)	- Showing the operation procedure to scrub nurses to enhance situation awareness	- Head mounted display; - Mild headache - Mild dizziness - Mild eye fatigue	- Use of Head-Mounted Display by scrub nurses could facilitate their understanding of operation procedure.
23	Wüller et al. 2018 Germany	- Pilot study, qualitative methods - explore situational change and further use cases for AR in nursing	b) Design science research method c) semi-structured interviews (*n* = 5)	- Wound care management with smart glasses	- Smart Glass	- benefits regarding accuracy of wound documentation are expected - communication with patient was experienced as more challenging

### Use cases

A majority of the studies did not describe the methods used for case identification, but three studies described them in detail. One publication used qualitative methods to identify relevant use cases [27], and one publication used quantitative methods [28]. One study mentions the combination of a literature review and interviews for use case identification [29]; however, the remaining twenty publications do not describe any methods used for use case identification.

The use case studies included here can be separated into the fields of nursing education [28, 30–39] and clinical settings [27, 29, 31, 40–47].

After use case identification, the process of requirements elicitation followed. Among the identified studies, the requirements elicitation was described with varying levels of detail. These varying levels are as follows: methods used for software development, the use of less standardized methods and no explicit methods used for requirements elicitation. In regards to software development, user-centred design [29, 44] and design science research methods [39, 47] are mentioned. In regards to less standardized methods, interviews and shadowing [27], the inclusion of an experienced nurse [42], iterative design and working ground [45], and analysing training sessions [32] were used.

Many of the studies without a method of requirements elicitation did not require one as no new applications were developed. The innovative aspect was the usage of an existing application in a new field [30, 31, 33, 40, 43]. Furthermore, eight studies did not describe their performed requirements elicitation [28, 34–38, 41, 46, 48, 49].

### Evaluation

Sixteen of the publications reviewed here performed evaluations with different aims. Most of them were broadly defined, e.g., Schneidereith states that the aim of her evaluation was describing errors in medication and administration, whereas Grünerbl et al. listed a range of questions to evaluate. Some studies focus on evaluating one specific application, task or device [30, 31, 36, 37, 40, 43, 46, 47], while others aim to get evidence of the type of task or device suitable for applications [29, 32, 34, 35, 41, 42]. In addition, two publications focus on providing general insights by evaluating specific applications [27, 33].

Conversely, seven studies did not conduct any evaluations. Two of them describe only the design process of an application [39, 44], whereas the other focuses on the development of a guideline instead of an application [45]. Additionally, some articles focus on a broad overview regarding the use of AR [48, 49]. One study refers to another publication describing the evaluation [28], while one article describes its prototype without an evaluation [38].

A variety of evaluation methods are described in these publications. Both qualitative [29, 30, 47] and quantitative methods [28, 32, 35, 40, 43, 46] were used separately, but the majority of studies relied on a mixed methods approach [27, 31, 33, 34, 36, 37, 41, 42].

Each of the included publications describes the potential use of AR in nursing. Different advantages of using AR are mentioned including hands-free usage of a device [27], reduction in the anxiety of patients [40, 48], time savings [33, 46], individual visualization [28], easy information retrieval [29, 32, 33, 41, 42], observation from different perspectives [30, 37, 43], increased accuracy of documentation [47], and support of simulations [31, 34, 36, 39].

There are possible negative effects including the need for attention on the device [29]. This may be critical as it takes away focus from the patient [33]. Furthermore, communication with the patient may become challenging [47]. In addition, due to concentration on the system, other hints may be missed [31].

### Devices used

Table 3 the devices used to identify technical challenges. Most of the studies used a Smart Glass, but some used a Smart Watch, a Head Mounted Display, a Helmet Mounted Display, a Smartphone or a Tablet. Some combined different devices, and one did not specify the device used. Technical challenges were identified during the use of each device.

**Table 3:** Devices used and technical challenges

Device	Technical challenges
Smart Watch	- Energy consumption and screen size [55] - No technical challenges described [32]
Smart Glass	- Image quality [29, 44] - Screen size [45] - Network connectivity [27, 30] - Speech recognition [27, 44] - No technical challenges described [28, 32, 37, 47, 49]
Tablet	- Response times, scanning, internet connection, stability of application [34] - Application has to be dependable [36] - No technical challenges described [40]
Helmet Mounted Display	- Problems with a bulky helmet, problems with the marker, and parallax vision [42]
Head Mounted Display	- Marker problems [41] - Mild headache, mild dizziness, and mild eye fatigue [43]
AR Headset	- Connectivity issues, lack of experience with system, and battery life [31]
Smart Phone	- Response time, incompatibility, scanning, internet connection, stability of application [34] - No technical challenges described [35, 46]
Not specified	- Immersion sickness, over-reliance on technology, lack of attention to surroundings [48]

## Discussion

The number of empirical studies focusing on AR in nursing is relatively modest. Existing studies focus on evaluating prototypes with a variety of methodological approaches instead of long-term field trials. Thus, identifying an evidence-based practice for implementing AR in nursing remains a goal for future research. Nevertheless, our review has revealed some important insights.

The increasing number of publications on AR in nursing in the past few years (only five before 2015) shows the growth of the field.

### Principal results

Identified use cases focus on specific fields of use, and use case identification and requirements elicitation are often not described in detail. In addition, we determined that the results of studies evaluating AR in nursing were predominantly positive; however, several technical challenges are described for most of these devices. Moreover, most applications could be identified as prototypes in an early stage of implementation. The settings in which the studies operated are noteworthy. While twelve studies can be grouped into a broad clinical setting with the variation in use cases, eleven studies are set in the field of nursing education.

We found that many studies focused on obtaining knowledge on the applications developed instead of the effects of technology inclusion on nursing. No studies questioned the clinical relevance of their results. Considering context while evaluating applications would be another goal for future research. This could be achieved through performing field trials for longer periods of time.

For future development trends we infer that further technological advances will lead to new use cases for AR in nursing. Which may be developed rapidly and need to be investigated in question of added value and impact, afterwards.

### Comparison with prior work

Although the inclusion of values into technology development [50] and the design of technologies in nursing are needed and the “unreflective handling” of technology in nursing is occasionally criticized [51, 52], our review shows that values are only barely recognized for designing and evaluating AR in nursing. Methods such as Value sensitive design may integrate values to shape the design of technology [50]. According to the literature, these methods are not currently being used in the development of AR in nursing.

Implementing technology into a new field when there is not a demand for it is called the technology-push approach. This approach is criticized as it introduces technology without any real need and may not solve problems [53]. Therefore, we argue that careful evaluation is especially reasonable in these cases.

Furthermore, our results show that the evaluation methods used in the literature did not include the whole context of technical implementation. Some authors claim that the context of technological implementation is important [54], but most publications did not agree or take the effects of these evaluated technologies into account.

For these reasons, we conclude that future publications should focus on performing long-term evaluations to take framing conditions and the long-term consequences of AR use into account. It is to mention that AR is still in a process of various technical improvements which can only be predicted to some extent. We argue though, that long-term evaluations of newly implemented or soon to be implemented applications and devices will be beneficial to further works. As some of the emerging findings will be transferable onto technical improvements yet to come, it might also prove useful to explore a broader range of evaluations of AR applications in different contexts. On the one hand this could be additional studies from the field of healthcare with and without mentioning nursing. On the other hand it could be useful to take different fields without any direct link to the healthcare sector such as design and manufacturing [6] or maintenance and logistics [2] into account. This could allow to learn from possibly made mistakes in other areas as well as to get a more differentiated view on problems specific to the field of nursing.

### Limitations

This review provides information for future research regarding AR in nursing; however, our findings are limited and must be interpreted with caution. First, we identified a relatively small number of studies that focused on AR in nursing. Second, we did not assess the quality of the studies included because this is a scoping review [24]; thus, studies with varying quality are included, and the results may have limited reliability. Third, negative results regarding AR in nursing may have been missed due to publication bias. Forth, studies did not focus on specific elements of nursing and did not focus on long-term implementations.

## Conclusions

Our results show that the methods for identifying use cases and evaluating applications differ between studies. Furthermore, the devices used vary from smart glasses to tablets and smart watches. Many of the reviewed studies evaluated the use of Google glass. These results show that the current design and evaluation of AR for nursing are conducted without taking values into account. Furthermore, the evaluations did not consider framing conditions.

Our results are important and informative for the nurses and technicians who are associated with the development of new technologies. They can use this review to reflect on their own design of use case identification, requirements elicitation and evaluation.

## Abbreviations

AR: Augmented reality
GG: Google glass
